# Rebuilding Trust on Routine Immunization in Era of COVID-19 Fear–Role that Civil Society Organizations can Play Hands-in-Hand with Immunization Program

**DOI:** 10.3389/phrs.2021.1603989

**Published:** 2021-06-02

**Authors:** Ameer Muhammad, Daniyaal Ahmad, Eleze Tariq, Yasir Shafiq

**Affiliations:** ^1^ VITAL Pakistan Trust, Karachi, Pakistan; ^2^ Medical College, Aga Khan University, Karachi, Pakistan

**Keywords:** COVID-19, routine immunization, CSOs, demand, improving coverage

To the Editor,

The COVID-19 pandemic has drastically impacted the health and socioeconomic condition of this symbiotic globe [1]. The aforementioned impact as well as the one on quality of life has been much more devastating for developing countries, such as the ones supported by the Global Alliance for Vaccines and Immunization (GAVI) [4] All GAVI-supported countries pledged to achieve 100 percent of immunization but one in every 10 children globally still has no access to vaccines, leaving millions of zero-dose children behind [6]. Where the supply-side in these countries is struggling to deliver vaccination services after COVID-19, the demand is facing “double burden” now. Communities often harbor lots of “myths and misconceptions” pertaining to vaccines and vaccination services [7]. The exacerbation of existing fear within communities to access vaccination services and anxiety among frontline health workers to safely deliver vaccination present as key hurdles, reflected by a much smaller number of children being vaccinated in this period [8–10]. The consequences of this have been compounded by restrictions on movement, suspension of public transport, and concerns about exposure to COVID-19. At the same time, the health workforce is absent from their duties either due to travel restrictions, self-quarantine, sickness, lack of personal protective equipment (PPE) and concerns about COVID-19 exposure [11, 12].

There is a resultant visible impact seen on immunization services such as the 52.8% decrease in immunization visits seen in the Sindh province, Pakistan. This translated into around 2,734 missed children daily only in Karachi, the most populous city of Sindh province. These missed children are at the highest risk of developing VPDs [13]. Many immunization campaigns like “polio and zero dose” were or are still suspended in countries including Pakistan, and bring about a risk of spread of these VPDs and consequent morbidity and mortality [14, 15].

Currently, countries are lifting lockdown restrictions and restoring the health services. However, due to the unpredictability of the situation and decrease in vaccination coverage, exceptional strategies are needed. Expanded Program on Immunization (EPI) alone cannot compensate for the loss of missed children so far and months of effort are required to reach the point where we were before COVID-19. Integrated systems and synergy across the partners are a need of the hour. In some semblance of hope, Pakistan has an existing infrastructure of CSOs which operate as the key players in delivering vaccination–playing major roles at both the supply-as well as the demand-side [16]. The role of these organizations in the response is integral as they are the last-mile players to reach the community’s needs, playing a pivotal role in containing endemic illnesses at the community level, averting cases through community mobilization and awareness, and acting at the front line in case detection and referrals. They also advocate with and hold the government, private sector, and development partners accountable for delivering to the most vulnerable [17, 18].

CSOs are thus potential frontline community players to delivering key messages related to COVID-19 and the emerging risk of VPDs in communities [19]. CSOs can fill the current gaps created in service provision and help in reconstructing the trust which vaccination services may have lost in terms of demand in hard-to-reach, vulnerable, and marginalized populations [20, 21]. They usually operate as an independent entity, granting them adjustable and contextual flexibility when working for the communities during the COVID-19 crisis. With having appropriate institutional support and already being trusted partners of the communities through cross sectoral intervention and integrated approaches, they harbor incredible potential in fulfilling this role [22–24].

The COVID-19 pandemic is an extreme condition and warrants the need of a supportive system for routine immunizations across the globe. The synergy between CSOs and the government needs to be established through the recognition of the contribution which these players can provide during this critical time and help the EPI programs to cover its losses. There are platforms available such as “Pakistan CSOs Coalition for Health and Immunization (PCCHI)”-a network of around 80 organizations working together on health and immunization in Pakistan. This forum has great power to aid in community mobilization, research, advocacy, awareness raising, demand generation, monitoring, and service delivery [25]. This unified action of formal CSO engagement and cohesive approach can help us reach those children who were missed, and supply them with the appropriate dose of vaccine, possibly preventing a lot of non-COVID-19 deaths which are attributable to VPDs. Data has suggested that; “For every one excess Covid-19 death attributable to SARS-CoV-2 infections acquired during routine vaccination clinic visits could be around 140 (37–549) and these deaths in children may be prevented by sustaining routine childhood immunization in Africa” [26]. Due to the significant impact in saving lives, there is a strong and urgent need for building these engagements and taking CSOs onboard who can work with the EPI program hand-in-hand, for the betterment of our communities and our children.

## Abbreviations

CSO, civil society organization; EPI, Expanded program on immunization; GAVI, Global alliance for vaccines and immunization; PCCHI, Pakistan CSOs coalition for health and immunization; VPD, vaccine preventable diseases.
